# Very late Magmaris scaffold restenosis: a 6-year serial optical coherence tomography case report

**DOI:** 10.1093/ehjcr/ytae344

**Published:** 2024-07-17

**Authors:** Jens Trøan, Kirstine Nørregaard Hansen, Manijeh Noori, Jens Flensted Lassen, Lisette Okkels Jensen

**Affiliations:** Department of Cardiology, Odense University Hospital, Odense, Denmark; Department of Cardiology, Odense University Hospital, Odense, Denmark; Department of Cardiology, Odense University Hospital, Odense, Denmark; Department of Cardiology, Odense University Hospital, Odense, Denmark; Department of Cardiology, Odense University Hospital, Odense, Denmark

**Keywords:** Bioresorbable scaffolds, Scaffold failure, Scaffold restenosis, Optical coherence tomography, Plaque rupture, Case report

## Abstract

**Background:**

Bioresorbable scaffolds (BRS) have been proposed as an alternative to drug-eluting stents (DES), offering radial support during the early phases of healing, while potentially reducing the risk of long-term complications. A magnesium-based BRS (MgBRS) has shown promising results after implantation. However, there is a lack of knowledge regarding the long-term outcomes.

**Case summary:**

A 62-year-old man with hypertension, dyslipidaemia, family history of ischaemic heart disease, and previous myocardial infarction, presented with non-ST-elevation myocardial infarction (NSTEMI). Six years prior, he also had a NSTEMI and a mid-left anterior descending artery (LAD) lesion was treated with a 3.0/25 mm MgBRS. Post-implantation optical coherence tomography (OCT) revealed proximal edge dissection, and a second MgBRS 3.0/15 mm was implanted. Optical coherence tomography of the scaffold-treated segment was performed after 6 and 12 months with no sign of restenosis. The current angiogram showed a restenosis in the previously MgBRS-treated segment in LAD. Optical coherence tomography showed a plaque rupture in a thin cap fibro-atheroma and scaffold remnants. The lesion was pre-dilated and stented with a 3.0/20 mm DES and post-dilated with a 3.5 mm non-compliant balloon.

**Discussion:**

Most cases of late scaffold failure showed acquired mal-apposition, which also can be related to the degrading process, or uncovered struts, none of which were seen in our case at 6 or 12 months. This case represents an insight into the vascular healing and potential mechanisms for failure of the MgBRS, with serial OCT recording at implantation, and after 6 months, 12 months, and 6 years.

**Learning points**

- Bioresorbable scaffolds were proposed to reduce long-term complications compared with drug-eluting stents. However, they still potentially compose a risk of scaffold failure several years after implantation.
- Possible mechanisms of very late magnesium-based bioresorbable scaffold failure are scaffold mal-apposition or under-expansion, discontinuation of antiplatelet therapy, neoatherosclerosis, and progression of coronary artery disease.
- Optical coherence tomography recordings give valuable insight into the possible mechanisms for very late scaffold failure and should be used when assessing patients presenting with very late scaffold failure.

## Introduction

Bioresorbable scaffolds (BRS) have been proposed as an alternative to drug-eluting stents (DES), offering radial support during the early phases of healing, while potentially reducing the risk of long-term complications and facilitating future interventions.^1^ The sirolimus-eluting magnesium-based BRS (MgBRS) Magmaris (Biotronik AG, Bülach, Switzerland) has shown promising long-term results after implantation.^2–4^ However, some studies have shown lumen reduction following implantation with early generation MgBRS.^5–7^

The first-generation Absorb scaffold showed good results after 1 year but had significantly higher device thrombosis at 3 years compared with DES.^8^ In the BIOSOLVE-IV study, the long-term outcomes up to 2 year of the MgBRS found promising results, with 6.8% target lesion failure (TLF).^4^ However, long-term outcome data of the MgBRS are limited.

In this case, we describe a very late MgBRS restenosis in a patient treated with the MgBRS, where serial optical coherence tomography (OCT) recordings were available from implantation and at 6 months, 12 months, and 6 years.

## Summary figure

**Figure:**
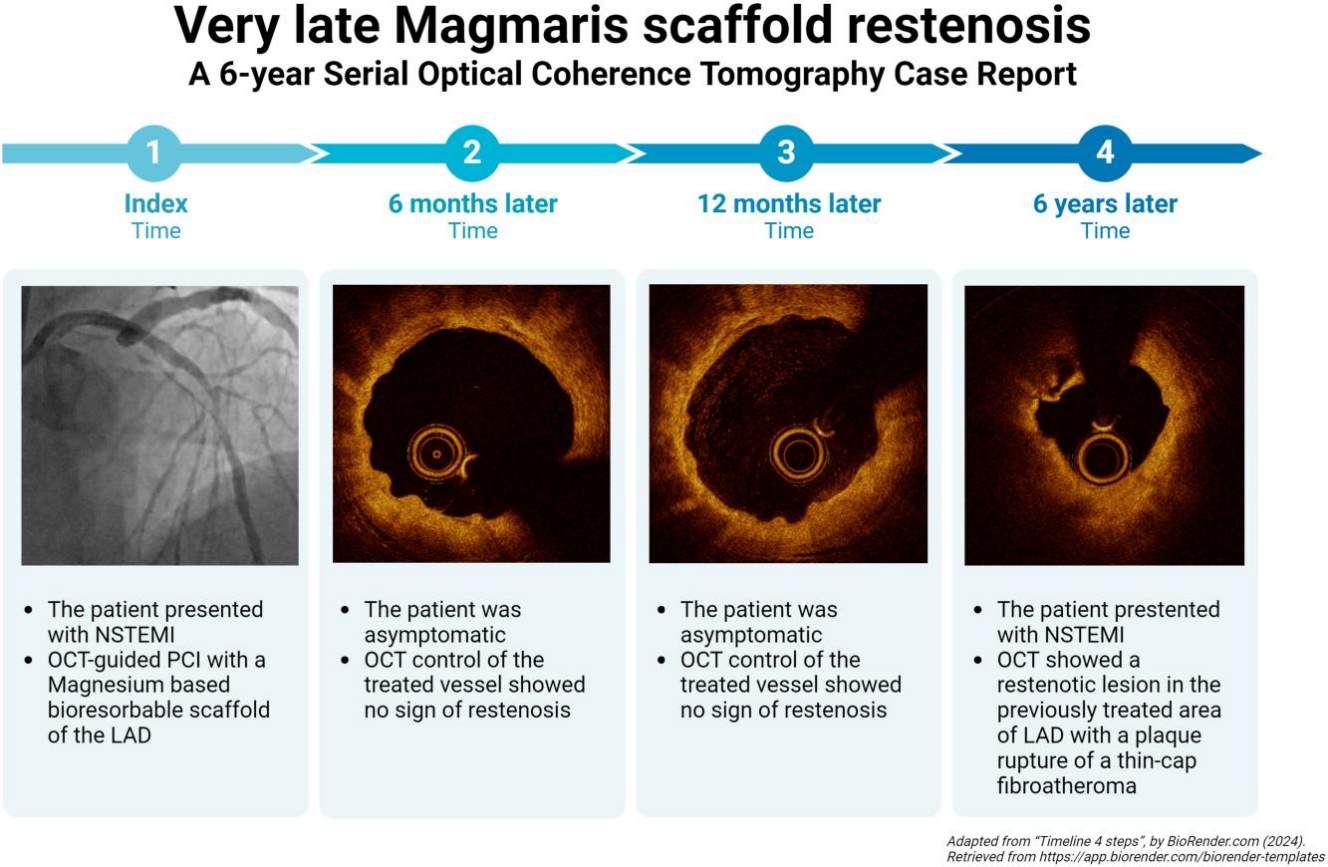


## Case presentation

A 62-year-old man with hypertension, dyslipidaemia, family history of ischaemic heart disease, and previous myocardial infarction presented with non-ST-elevation myocardial infarction (NSTEMI).

Six years prior, he presented as well with NSTEMI, and a mid-left anterior descending artery (LAD) lesion (Supplementary material online, *Video S1*) was treated with OCT-guided percutaneous coronary intervention (PCI) with a 3.0/25 mm MgBRS after 1:1 pre-dilation. Post-implantation OCT revealed proximal edge dissection, and a second MgBRS with intending overlap 3.0/15 mm was implanted. Post-dilation with a 3.5 mm non-compliant balloon was performed. Final OCT verified a sealed edge dissection and two well-apposed scaffolds without overlap, but an uncovered gap of 1.2 mm. Implantation of the MgBRS was performed using the recommended ‘4P strategy’^9^ with (i) patient and lesion selection: a patient with NSTEMI and non-calcified lesion; (ii) proper sizing: using imaging-guided PCI; (iii) pre-dilatation; and (iv) Post-dilation. The patient was treated with ticagrelor 90 mg twice a day for 12 months and aspirin 75 mg and statin daily livelong.

Due to enrolment in a randomized clinical trial, coronary angiogram and OCT of the scaffold-treated segment were performed after 6 and 12 months with no sign of restenosis (Supplementary material online, *Video S2*).

**Figure 1:**
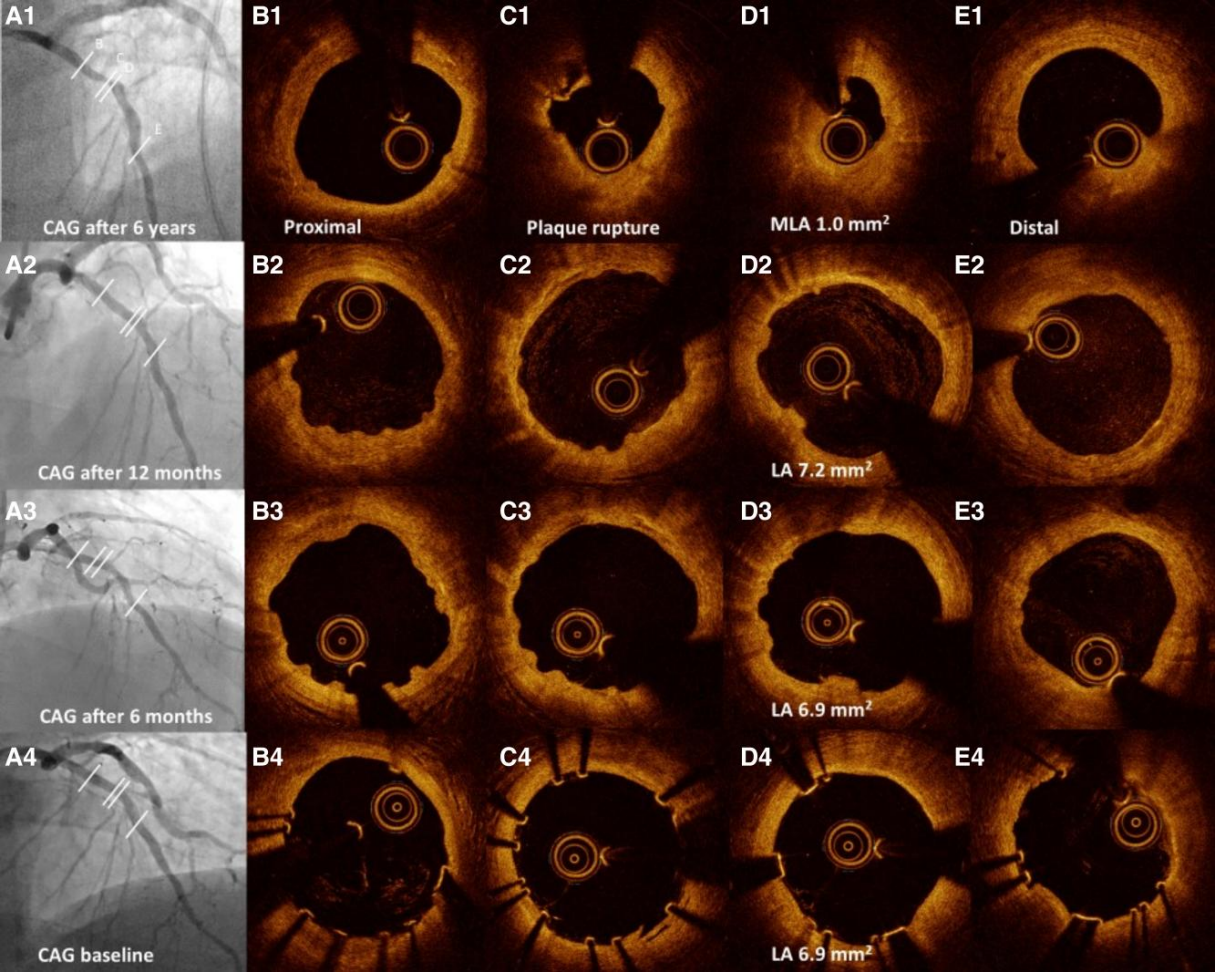
(*A*) Coronary angiogram, (*B*) optical coherence tomography of proximal scaffold, (*C*) optical coherence tomography of plaque rupture segment, (*D*) optical coherence tomography of minimal lumen area, and (*E*) optical coherence tomography of distal segment at 1 = restenosis event, 2 = 12-month follow-up, 3 = 6-month follow-up, and 4 = baseline event. CAG, coronary angiogram; LA, lumen area; MLA, middle lumen area.

The angiogram 6 years after implantation showed a restenosis in the previously MgBRS-treated segment in the mid-LAD (*Figure 1A1*; Supplementary material online, *Video S3*). Optical coherence tomography showed a plaque rupture in a thin cap fibro-atheroma (*Figure 1C1*) 3.0 mm proximally to thrombus formation and minimal lumen area (MLA) of 1.0 mm^2^ (*Figure 1D1*). No uncovered MgBRS struts, but a few struts remnants, were found (*Figure 1B1* and *E1*). The patient was compliant with antiplatelet therapy at the time of the restenotic event.

Retrospective analyses of previous OCT recordings, using BRS proximal marker and side branches as markers, showed no signs of mal-apposition, under-expansion, or scaffold fracture at the site of 6-year follow-up MLA (*Figure 1D4*) in the post-implantation OCT. The 6-year MLA was not in relation to the edge dissection or scaffold gap, and the treated area was without calcification.

The OCT pullbacks at follow-up after 6 and 12 months revealed no sign of late lumen loss (*Figure 1D2* and *D3*). Struts remnants were found after both 6 and 12 months (*Figure 1B2* and *B3* and *E2* and *E3*). There was no sign of scaffold fracture, acquired scaffold mal-apposition or under-expansion at 6 and 12 months (*Figure 1C2–D2* and *C3–D3*). The scaffold edge dissection from implantation showed complete healing at 6 months.

The MgBRS restenotic lesion was pre-dilated and stented with a DES and subsequently post-dilated. The patient was prescribed with lifelong aspirin and 12-month prasugrel, and he was free from any cardiac symptoms 1 year after the DES implantation.

## Discussion

In clinical practice, it is recommended that the procedure and implantation of a MgBRS are following the guidelines of the ‘4P strategy’: (i) patient and lesion selection, (ii) proper sizing, (iii) pre-dilatation, and (iv) post-dilatation.^9^ Despite following the guideline closely, a very late restenosis occurred 6 years after implantation. In the following, potential mechanisms and possible reasons will be discussed.

Clinical presentation has an impact on scaffold failure in the Magmaris stent. In BIOSOLVE-IV, patients who presented with NSTEMI, like in our case, were significantly associated with higher rates of TLF,^4^ meanwhile two cases have been reported of MgBRS late restenosis in stable disease.^10,11^

Scaffold mal-apposition and under-expansion may be important factors for both early and late scaffold failure.^12^ Due to the degrading process of the BRS, cases have been reported with late acquired mal-apposition giving rise to stent thrombosis and scaffold failure, due to scaffold dismantling.^10^ Furthermore, in cases describing very late scaffold thrombosis of the Absorb BRS, mal-apposition and under-expansion were found to be important factors.^13,14^ In our case, mal-apposition or under-expansion were not seen at implantation, 6 months, 12 months, or after 6 years. In addition, the term scaffold failure is debatable in patients presenting very late, where the scaffold is assumed to be re-absorbed. However, in our case, strut remnants were seen on OCT, challenging the view of a fully absorbed scaffold in all patients presenting very late.

Discontinuation of antiplatelet therapy is a potential reason for scaffold failure^12^ and has been reported in several cases of very late scaffold thrombosis with the Absorb BRS.^13,14^ In our case, the patient was compliant for 1-year dual antiplatelet therapy and was on aspirin at the time of the restenotic event.

Progression of coronary arterial disease is another potential mechanism for the observed late scaffold restenosis. The patient had aggressive coronary arterial disease with diffuse atherosclerosis of the left circumflex artery and a chronic total occlusion of the right coronary artery. In the Absorb BRS, it has been described a formation of a signal-rich layer on OCT, potentially encapsulating the atherosclerotic process and protecting the vessel.^15^ No such layer was seen in our case. It is possible that the faster degrading process of the MgBRS might prevent this layer from forming and thereby potentially having an increased risk of very late restenosis as a result of increased progression of the coronary artery disease compared with the Absorb scaffold.

## Conclusion

Six years after implantation of a MgBRS, restenosis with plaque rupture was detected on OCT. No sign of restenosis, scaffold fracture, or scaffold mal-apposition was found on OCT during serial 6- and 12-month follow-up.

## Supplementary Material

ytae344_Supplementary_Data

## Data Availability

Data regarding this case are available on reasonable request.

## Lead author biography

Dr Jens Trøan graduated from the University of Southern Denmark in 2023. He is a medical doctor and clinical researcher under the guidance of Professor Lisette Okkels Jensen MD PhD Dr.med at the Department of Cardiology, Odense University Hospital. His areas of interests are invasive coronary physiology, PCI, and clinical trials

## Supplementary material

**Consent:** Written consent for the submission and publication of this case was obtained from the patient in line with the COPE guidelines.

**Funding:** None declared.
